# Targeting Leukemia Stem Cell-Niche Dynamics: A New Challenge in AML Treatment

**DOI:** 10.1155/2019/8323592

**Published:** 2019-08-07

**Authors:** Paolo Bernasconi, Oscar Borsani

**Affiliations:** University of Pavia, Department of Molecular Medicine, Fondazione IRCCS Policlinico San Matteo, Pavia, Italy

## Abstract

One of the most urgent needs in AML is to improve the disease cure rate as relapse still occurs in 60–80% of patients. Recent evidence suggests that dismal clinical outcomes may be improved by a better definition of the tight interaction between the AML cell population and the bone marrow (BM) microenvironment (“the niche”); the latter has been progressively highlighted to have an active role in the disease process. It has now been well established that the leukemic population may misinterpret niche-derived signals and remodel the niche, providing a shelter to AML cells and protecting them from the cytotoxic effects of chemoradiotherapy. Novel imaging technological advances and preclinical disease models have revealed that, due to the finite number of BM niches, leukemic stem cells (LSCs) and normal hematopoietic stem cells (HSCs) compete for the same functional areas. Thus, the removal of LSCs from the BM niche and the promotion of normal HSC engraftment should be the primary goals in antileukemic research. In addition, it is now becoming increasingly clear that AML-niche dynamics are disease stage specific. In AML, the niche has been linked to disease pathogenesis in the preleukemic stage, the niche becomes permissive once leukemic cells are established, and the niche is transformed into a self-reinforcing structure at a later disease stage. These concepts have been fostered by the demonstration that, in unrelated AML types, endosteal vessel loss occurs as a primary AML-induced niche alteration, and additional AML-induced alterations of the niche and normal hematopoiesis evolve focally and in parallel. Obviously, this endosteal vessel loss plays a fundamental role in AML pathogenesis by causing excessive vascular permeability, hypoxia, altered perfusion, and reduced drug delivery. Each of these alterations may be effectively targeted by various therapeutic procedures, but preservation of endosteal vessel integrity might be the best option for any future antileukemic treatment.

## 1. Introduction

In AML, there is an urgent need to develop more effective treatments as the current standard of care chemotherapy cures only 40–45% of younger and 10–20% of older patients [1]. The cytogenetic/genomic categorization of the disease has allowed the precise definition of distinct AML molecular subsets but has not translated into an increased number of drugs inspired by genomics. In addition, current evidence suggests that AML clinical outcomes might be improved not only by more selective and better tolerated treatments that target the AML cell population but also specifically through the development of novel strategies that target the interaction between the AML population and the BM microenvironment (“the niche”) whose critical role in AML has been progressively defined [2–4]. Despite the complex and sometimes conflicting data, various seminal studies have highlighted that normal hematopoietic stem and progenitor cells (HSPCs) and leukemic stem cells (LSCs) utilize bidirectional cross talk with their neighboring niche cells to create specific reciprocal dependencies [5, 6]. An early study reported that AML homing occurs around vessels of a specialized E-selectin and CXCL12 positive endothelium [7], two subsequent studies reported that leukemic engraftment occurs in areas enriched with osteoblasts [8, 9], and another has shown that LSCs are themselves able to generate walled-off abnormal niches [10]. In addition, an increase in microvascular density, altered innervation [11], and the loss of osteoblasts [12] were reported to occur in the AML niche, but no correlations with the disease stage or type were established. Instead, current intravital imaging and genetic mouse models have revealed that the interactions between malignant cells and the BM microenvironment are disease subtype and stage specific [13, 14], and this assumption has also been validated in AML. Osteoblasts halt the expansion of BCR-ABL CML-like disease but promote the propagation of MLL-AF9-driven AML [4]. The niche, which is necessary for leukemogenesis in a preleukemic stage, becomes permissive once leukemia is established [2], and it is transformed into a self-reinforcing leukemic compartment at a later disease stage [3]. However, the most relevant finding provided by intravital imaging and genetic mouse models is that the primary alteration induced by the AML cell population affects the vascular architecture and function of the niche [13, 15]. The loss of osteoblasts and the suppression of normal hematopoiesis occur at a later disease stage, and these events evolve focally and in parallel and end in niche collapse that is no longer reversible. These data are of pivotal relevance as they further improve our knowledge regarding the mutual cellular influences that allow the development of AML through the adjustment of secure signaling pathways involved in self-renewal, proliferation, survival, and immune evasion. More importantly, these vascular alterations that consist of increased vascular permeability, hypoxia, altered perfusion, and reduced drug delivery may become potential targets of novel candidate interventions that might restore the normal niche ecology making AML cells more susceptible to chemotherapy. This review focuses on possible interventions that might restore normal hematopoiesis and normalize niche function.

## 2. Strategies to Dislodge LSCs from Functional Niches

The demonstration that the number of functional niches within the BM is limited and that niche occupancy is the limiting factor in transplantation of normal HSPCs is the basis for the notion that LSCs and HSCs might share and compete for the same BM niches [8, 9, 16, 17]. In addition, functional niches within both the BM and extramedullary compartments may protect LSCs from the toxic effects of chemotherapy and the graft-versus-leukemia (GvL) effect by creating “sanctuary sites” that provide LSCs with survival signals and improve immune escape by limiting antileukemic T-cell responses [18]. Colmone et al. were the first to propose the existence of a competition between HSCs and LSCs. In the BM of a severe combined immunodeficiency (SCID) mouse model with a pre-B acute lymphoblastic leukemia (ALL), they demonstrated that both normal HSCs and leukemic stem cells (LSCs) localize to specific vascular niches enriched with stromal cell-derived factor-1 (SDF-1)/CXCL-12 [7]. They also found that leukemic cells alter the BM microenvironment by creating malignant areas that prevent CD34 + cell engraftment through the release of stem cell factor (SCF). Thus, therapeutic targeting of SCF may increase the hematopoietic reserve and improve engraftment of allogeneic and autologous stem cells [10]. Subsequently, other studies reported that, in experimental mouse models, the cotransplantation of increasing doses of normal HSCs or cord blood hematopoietic stem/progenitor cells (CB-HSPCs) together with AML cells prolonged animal survival by inhibiting disease progression and resulted in distinct foci of either normal HSCs or AML cells in the early posttransplant period [19, 20]. In addition, in these cotransplantation models, high doses of normal HSPCs were able to decrease leukemic cell cycling, confirming the clinical observation that, in human transplantation, leukemia relapse is significantly lower in patients who receive high than in those who receive low CD34 + cell doses [21]. This result that does not seem to be due to a more robust graft-versus-leukemia (GvL) effect is provided by the higher CD34 + cell doses. These observations strengthened the idea that normal HSCs might effectively outcompete LSCs for niche occupancy especially after some particular cellular manipulations. The most impressive results were obtained when the infusion of CB-HPSCs occurred soon after (i.e., one hour after) the mobilization of AML cells, in comparison with infusions made one day after their mobilization. In order to explain these results, the potential return of AML cells to their BM niches was suggested based on the observation that, in the context of preestablished leukemic disease, poor CB-HPSC engraftment occurred. Other studies have hypothesized that the targeting of adhesion molecules overexpressed by AML cells (e.g., CXCR4, very late antigen 4 (VLA4, also known as *α*4*β*1 integrin), CD44, E-selectin, and CD98) together with the exploitation of the aforementioned competition between normal and leukemic stem cells for the same BM niches might be an effective therapeutic strategy in patients with minimal residual disease (MRD) at the time of hematopoietic stem cell transplantation (HSCT). Among the adhesion molecules responsible for normal and leukemic stem cell homing to BM niches, CXCR4 which binds to CXCL12 appears to be the most relevant one as it controls migration, mobilization, homing, and retention of LSCs within the BM and activates apoptosis that is prevented by an as-yet-undefined soluble factor produced by osteoblasts [22–24]. The CXCR4-CXCL12 axis activates PI3K/Akt and MEK/ERK pathways and downregulates microRNA let-7a. In AML, increased expression of CXCR4 on LSCs has been associated with a shorter survival and an increased relapse rate [25–27]. Thus, as demonstrated in xenograft models, targeting the CXCR4-CXCL12 axis might be an effective treatment strategy that would achieve more profound disease eradication by mobilizing LSCs [28, 29] from their “sanctuary sites” rendering them more susceptible to both the toxic effects of chemotherapy and the antileukemic T-cell response [28, 30]. This explanation provides the rationale for combining the principal inhibitor of the CXCR4-CXCL12 axis (i.e., plerixafor) with conventional chemotherapy [31]. Phase I/II clinical trials based on this combination have provided beneficial results [32]. Importantly, the addition of plerixafor to the myeloablative regimen for allogenic HSCT for AML patients in their first complete remission is a safe and well-tolerated therapeutic procedure [33]. Thus, further studies in larger cohorts are warranted to investigate the impact of plerixafor on the relapse rate and survival of transplanted patients.

Mobilization of LSCs might also be achieved by targeting the VCAM1/VLA4 axis, which mediates the binding of AML cells to endothelial cells with the development of a quiescent leukemic cell phenotype. These AML cells are physically and functionally integrated within the endothelium but may still cause relapse [34]. However, disrupting the VCAM1/VLA4 axis may be effective only in patients with minimal residual disease, whereas in those with active disease, this strategy needs to be combined with conventional chemotherapy. In addition, natalizumab, a humanized VLA4 monoclonal antibody, which causes prolonged HSC mobilization [35], has limited clinical utility because it may cause JC virus-associated progressive multifocal leukoencephalopathy [36]. E-selectin is also involved in AML cell binding to the vascular niche with activation of Wnt signaling and might be another potential target of mobilizing treatments. AML cell binding to E-selectin is associated with increased localization of leukemic cells within the BM niche and their increased survival during chemotherapy. Thus, clinical trials with GMI-1271, a small E-selectin inhibitor, which in a mouse xenograft model enhanced the effects of chemotherapy, are currently ongoing [37]. In addition, leukemic cell binding to endothelial cells is also increased by the glycoprotein CD98 that binds integrin *β*1. Recent *in vitro* studies have revealed that a CD98 antibody significantly reduces adhesion and the colony-forming ability of primary AML cells, and in experimental murine models, it significantly reduces the AML tumor burden [38].

Sympathetic nervous system (SNS) neuropathy is another mechanism that in the MLL-AF9 AML model may lead to leukemia progression [1]. These leukemic cells decrease the density of the SNS network through the alteration of *β*2 adrenergic signaling with loss of niche quiescence. In particular, this loss is due to the expansion of mesenchymal stromal progenitor cells committed to osteoblastic differentiation and the reduction of arteriole-associated NG2+ niche cells. Thus, a potential therapeutic approach could be to circumvent this *β*2 adrenergic receptor damage with receptor agonists. However, to date, these last drugs have provided contradictory results.

**Figure 1:**
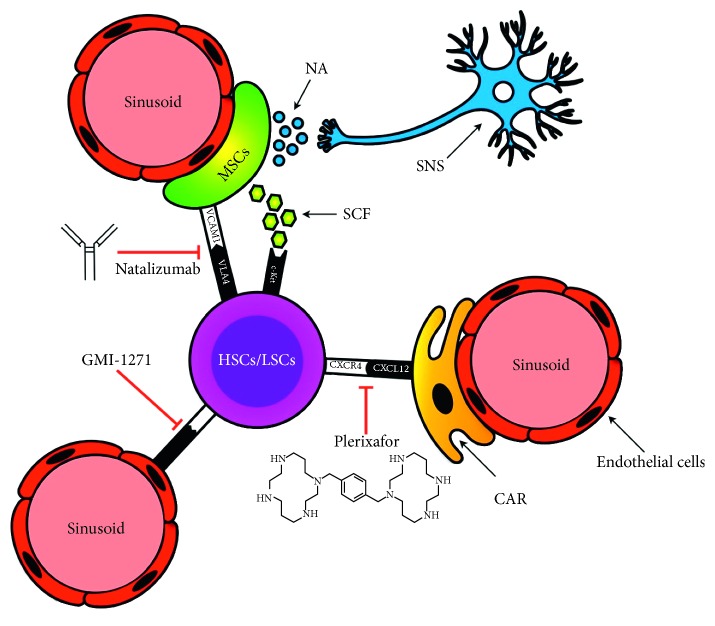
Bone marrow vascular niche. The vascular niche is composed of sinusoidal endothelial cells and mesenchymal stem cells (MSCs), both of which express adhesion molecules such as VCAM1, which bind to the corresponding receptor VLA4 (an *α*4*β*1 integrin) expressed on both HSCs and LSCs, and the soluble stem cell factor (SCF), which binds c-Kit to the surface of HSCs and LSCs. Natalizumab, an anti-VLA-4 monoclonal antibody, acts by preventing the VLA4-VCAM1 interaction. CXCL-12 is produced mainly by CXCL-12-abundant reticular cells (CARs). The binding of CXCL-12 to CXCR4 on HSCs and LSCs plays an important role in HSC and LSC homing and retention within the bone marrow. Plerixafor, which acts by inhibiting this link, promotes the HSC/LSC mobilization from the bone marrow vascular niche. E-selectin is expressed on endothelial cells and is involved in HSC/LSC retention within the bone marrow vascular niche through its interaction with sialylated carbohydrate expressed on both the HSC and LSC surfaces. GMI-1271, which is an E-selectin inhibitor, promotes HSC and LSC displacement by weakening this link. Finally, a close interaction between MSCs and adrenergic fibers has also been demonstrated. Release of noradrenaline by the sympathetic nervous system (SNS) induces metalloproteinase expression and activity, which then act to cleave other adhesion molecules (CXCR4, VLA4, VCAM1, and SCF), thereby promoting HSC release from the bone marrow [39, 40].

The BM vascular niche and the major interactions between its cellular components are summarized in Figure 1; the mechanisms by which different drugs mobilize HSC and LSC are also presented.

## 3. Targeting Hypoxia

**Figure 2:**
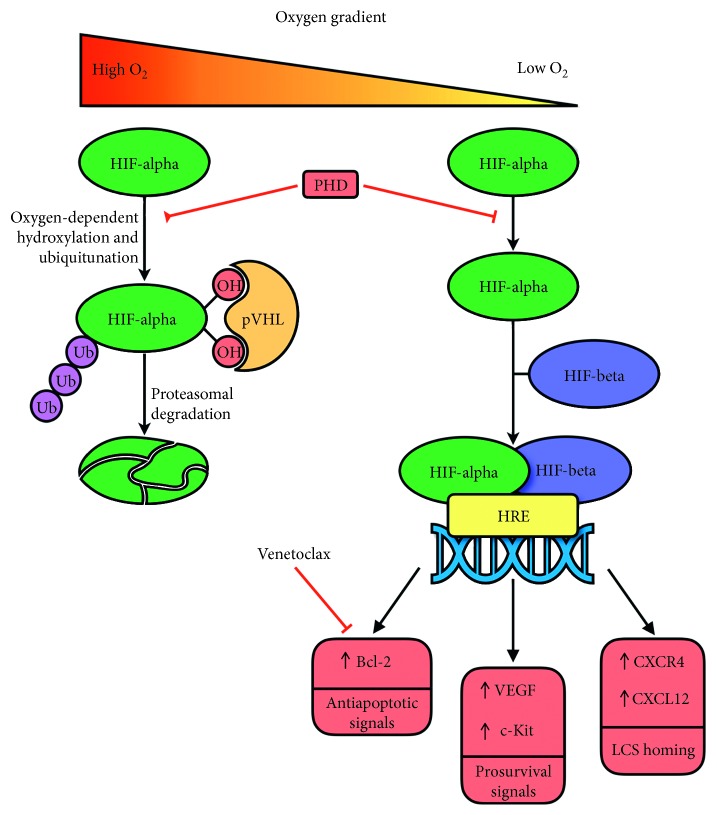
Role of hypoxia in leukemic stem cell maintenance. Under normoxia conditions, the alpha-subunit of hypoxia inducible factor (HIF-alpha) undergoes an oxygen-dependent hydroxylation by the enzyme prolyl hydroxylase (PHD). The hydroxylated form of HIF-alpha is recognized by the von Hippel–Lindau protein (pVHL); this interaction allows for ubiquitination and proteasomal degradation of HIF-alpha. Under hypoxic conditions, the hydroxylation and subsequent ubiquitination and degradation of HIF-alpha do not take place. Therefore, HIF-alpha is able to interact with the beta-subunit of hypoxia inducible factor (HIF-beta), forming a heterodimeric complex that can promote transcription of specific genes called hypoxia responsive elements (HREs). These genes encode for proteins with antiapoptotic (e.g., Bcl-2) and prosurvival (e.g., c-Kit and VEGF) functions. HREs also encode for CXCR4 and CXCL12, which play an important role in LSC homing and preservation. The ability of venetoclax, a potent Bcl-2 inhibitor, to induce LSC apoptosis is explained by the high level of Bcl-2 expression on LSCs.

Evidence coming from rat models has sufficiently demonstrated a close relationship between BM hypoxia and AML cells. In particular, it was demonstrated that AML cells preferentially infiltrate BM hypoxic areas, which expand on leukemia engraftment and disease progression [41]. In addition, AML cells from leukemic rats are more hypoxic than BM cells obtained from healthy rats [42, 43]. These hypoxic BM niches have a reprogrammed metabolism. A recent study showed that glycolysis was higher in the femurs of leukemic mice than in the femurs of healthy control mice. In addition, in samples from AML patients, leukemic blasts overexpressed genes defining a “hypoxia index” [44]. Interestingly, in these xenograft leukemia models, exposure to the hypoxia-activated prodrug TH-302 depleted hypoxic cells, prolonged survival, and reduced the leukemia stem cell pool especially when this hypoxia-activated prodrug was combined with sorafenib. More relevantly, TH-302 prolonged survival in a syngeneic AML model by eliminating residual hypoxic leukemic BM cells that had persisted after chemotherapy [44]. These results confirm that leukemic cells preferentially home to hypoxic niches where the compromised blood flow reduces their exposure to chemotherapy and immune effector cells [41–43, 45] and that these BM areas contain true quiescent and chemoresistant LSCs endowed with self-renewal capacity. Hypoxia activates the hypoxia-inducible factors 1*α* and 2*α* (Hif-1*α* and Hif-2*α*) and the PI3K/Akt/mammalian target of the rapamycin (mTOR) signaling pathway, which provides environmental prosurvival cues to leukemic cells. It was demonstrated that Hif-1*α* and Hif-2*α* through their interaction with the “hypoxia responsive elements” (HREs) of various genes (e.g., TGF*β*, c-Kit, FGF-2, VEGF, and Notch-1) may upregulate the expression of CXCR4 and CXCL12 on AML cells and endothelial cells, thereby promoting LSC maintenance and disease aggressiveness [46–48] (Figure 2). However, the most relevant effect of Hif-1*α*, which is stabilized by the AML-associated mutations of the isocitrate dehydrogenase (IDH) 1 and IDH2 genes, is to make leukemic cells completely dependent on mitochondrial oxidative phosphorylation to fulfill their low energy requirements as they are unable to employ glycolysis [49]. More importantly, Bcl-2, one of the most relevant mediators of mitochondrial respiration, is overexpressed in AML especially in patients with IDH1 and IDH2 mutations. Thus, Bcl-2 therapeutic targeting is expected to be highly effective in AML. This suggestion was confirmed by various studies. AML cells are exquisitely sensitive to the pharmacological blockade of Bcl-2 pathways since leukemic cells assemble the apoptotic machinery on the mitochondrial membrane kept in check by inhibitory Bcl-2 proteins [50]. In addition, it was demonstrated that ABT199 at nanomolar concentrations induced apoptosis in various AML cell lines in *in vitro* cultures of chemosensitive and chemoresistant AML stem and progenitor cells and inhibited leukemic progression in *in vivo* murine models [51]. A phase II clinical trial of venetoclax monotherapy in relapsed/refractory AMLs showed only modest activity; however, it identified the lack of myeloblast dependence on BCL-X_L_ or MCL-1 and the presence of IDH1/IDH2 mutations as the best predictors of response [52, 53]. In contrast, venetoclax was much more effective when combined with cytarabine and idarubicin [54], PI3K inhibitors [55], 5-azacytidine [56–58], and mTOR inhibitors [59]. Moreover, the combination of venetoclax with low-dose cytarabine which is currently being evaluated in a randomized phase III trial has up to now provided impressive results with a CR/CRi rate of 62% and a median overall survival of 11.4 months [60]. In addition, an ongoing phase Ib trial enrolling elderly patients with relapsed/refractory AML and employing venetoclax in combination with the MDM2 inhibitor idasanutlin has reported interesting results [61]. The rationale for combining these drugs was based on the demonstration that p53 activation through MDM2 inhibition reduces Ras/Raf/MEK/ERK signaling with GSK3*β* activation and MCL-1 degradation, hence overcoming venetoclax resistance [62]. Recent data have revealed that mitochondrial apoptosis can also be induced by glutaminase inhibitors that cause an increase in glutaminase levels and act synergistically with ABT compounds [63].

Hypoxia can also be targeted by hypoxia-activated prodrugs [41, 64]. TH-302 is one of these compounds that under hypoxic conditions release the DNA alkylating agent bromo-isophosphoramide mustard. A study reported that chemoresistant AML cells after exposure to TH-302 developed reduced HF-1*α* expression, DNA strand breaks, cell cycle arrest, and apoptosis [41, 64]. This observation provided the rationale for initiating phase I clinical trials on refractory AML, but these studies have achieved only modest results: most patients developed an early but transient cytoreduction that was not maintained until the next cycle and only two responded [65].

## 4. Targeting Leukemic-Stromal Cell Interactions

Additional treatment strategies aimed at hampering leukemic niche dynamics are based on the demonstration that leukemic cells may alter stromal cell activities and the BM microenvironment in order to favor disease development and progression. One of the mechanisms that AML cells use to remodel the niche is represented by exosome (i.e., membranous nanosized vesicles derived from the endocytic compartment) or microvesicle (larger vesicles produced by the shedding of the plasma membrane) release [66, 67]. miR150, miR210, and various transcripts contained within distinct exosomes/microvesicles may affect AML prognosis, treatment, and niche function by altering the behavior of bystander cells [67, 68]. Currently, Rab27a has been identified as one protein involved in exosome release and is a potential therapeutic target in experimental models. Recent data show that Rab27a knockdown reverses the ability of AML exosomes and AML cells to alter the BM composition of wild-type recipient mice. In particular, Rab27a knockdown eliminates the increase of SCA1+ CD146+ stromal cells and the block of osteoblast differentiation induced by *Dkk1* upregulation and significantly prolongs the survival of wild-type recipient mice [69]. Another intriguing therapeutic strategy might be to target the reciprocal contact-dependent transfer of functional mitochondria between mesenchymal stem cells (MSCs) and AML cells [70]. This transfer, which increases during chemotherapy providing a better leukemic cell survival rate, thanks to a 1.5-fold increase in energy production [70], might be targeted by cytochalasin, a finding that supports the potential involvement of nanotubes in this process [71]. Reciprocal mitochondrial transfer from AML cells to MSCs also occurs in leukemic patients [72]. These data, along with the observation that LSCs from tyrosine kinase inhibitor-resistant (TKI-res.) CML patients are also completely dependent on mitochondrial transfer, have led to the approval of tigecycline in combination with imatinib for TKI-res. CML [73]. Current evidence suggests that the AML cell metabolism also might be a promising treatment target. Recently, *in vitro* screening revealed that LSC growth is suppressed when these cells are cocultured with MSCs in the presence of lovastatin, a cholesterol-lowering drug, an effect that was not achieved when AML cells were cultured alone. In addition, infusions of cells obtained from LSC-stromal cell cocultures pretreated with lovastatin prolonged mice survival [74]. Even more recently, it was reported that etomoxir, an inhibitor of carnitine O-palmitoyltransferase 1 (CPT-1), a protein involved in the fatty acid oxidation (FAO) pathway, was able to sensitize an LSC subset to chemotherapy [75, 76]. These cells that express CD36 (a marker of poor prognosis) at high levels [77] are able to highjack the lipolytic process in the gonadal adipose tissue to fuel their metabolic needs and evade chemotherapy-induced apoptosis [78]. An LSC subset that was exposed to etomoxir was no longer able to use FAO, an alternative source of energy which interfered with BAX/BAK oligomerization in response to apoptotic stimuli [79, 80].

## 5. Maintaining Vascular Integrity

Various studies have reported that, in murine AML models and AML patients, the number of sinusoidal vessels within the BM central region (the so-called “BM microvessel density” (BM-MVD)) is increased [81–83]. It has been suggested that this alteration was induced not only by MSCs and megakaryocytes but also by the AML cells themselves [84, 85]. Nowadays, it is well known that leukemic cells may generate autocrine and paracrine loops through the release of vascular endothelial growth factor (VEGF) and angiopoietins 1 and 2 and the expression of their respective receptors [84, 86]. However, despite these data, AML patients have always had a disappointing response in clinical trials that employed antiangiogenic drugs, a result that can be explained by two very recent studies that identified endosteal vessel alterations as the primary events in AML pathogenesis [13, 15]. One study described a heterogeneous distribution of endothelial cells (ECs) with a reduction of those associated with sinusoids *versus* a significant increase of those associated with arterioles, a scenario that leads to the generation of several hypoperfused BM areas. At an early AML stage, these hypoxic areas, which appeared leakier than areas nonengrafted by AML cells, were localized near AML cells, whereas at a later disease stage, they were scattered throughout the entire BM [15]. Another study reported that the endosteum of the metaphysis, but not that of the diaphysis, was the main site of this vessel loss [13]. Moreover, with low BM infiltration (i.e., 5–15% of BOM infiltration), the appearance of the perivascular and endosteal stroma was normal; but with high infiltration, the appearance of these stromal areas was abnormal with depleted perivascular and endosteal stromal areas. In low-infiltrated areas, blood vessels appeared narrower than those of control mice and far from the endosteal surface. In contrast, in high-infiltrated areas, the formation and retraction of blood vessel sprouts were observed, and these lead to a less-effective angiogenic process. Finally, the detachment of ECs from the vascular wall leads to their accumulation within the vascular lumen and to their potential uptake by AML cells. EC depletion occurs as the primary event of AML-induced niche alterations and is followed by the concomitant loss of osteoblastic and HSCs. In addition, these alterations of the niche and normal hematopoiesis evolved focally and in parallel. Moreover, when the vascular niche had collapsed, no treatment strategy was able to induce its restoration [13]. Thus, it was suggested that the best therapeutic strategy is to prevent the AML-induced endosteal vessel loss in order to prevent AML progression due to the generation of BM hypoxic areas (real “sanctuaries” of chemoresistant cells) [9] and to favor leukemic cell targeting by cytotoxic agents [8, 87]. In order to achieve this goal, leukemic mice were treated with the iron chelator deferoxamine (DFO) that also enhances HIF-1*α* stability and activity, thereby promoting the expansion of endosteal vessels and increasing the number of HSCs within the trabecula-rich metaphysis. However, DFO did not modify the number of BM AML cells and did not improve the survival and disease progression rate unless it was combined with chemotherapy. Interestingly, recent evidence suggests that DFO by modulating reactive oxygen species (ROS) pushes the differentiation of leukemic blasts and normal BM precursors towards monocytes/macrophages. This observation points to a strong role of ROS in endosteal vascular niche remodeling [88]. In normal BM, NADPH oxidase 4 (NOX4), an endothelial isoform of NADPH oxidase, regulates the production of ROS and the activation of nitric oxide synthase 3 (NOS3) with the consequent generation of nitric oxide (NO) [15] in response to oxidative stress and hypoxia. The gene-encoding NOX4 is overexpressed by BM-derived ECs and in the BM of AML patients and patient-derived xenograft (PDX) mice. This NOX4 overexpression has been associated with NOS3 overexpression and ROS/NO overproduction. Of note, a persistent elevation of NO levels has been associated with an increased rate of treatment failure in AML patients. This evidence supports the results obtained with PDX mice, in which NO inhibition combined with Ara-C treatment reduced NOS3 activation, vascular leakiness, and BM hypoxia, leading to reduced AML progression in the BM and spleen and a “remission-like” phase longer than that of control mice. Furthermore, NO inhibition combined with Ara-C treatment was more efficient than chemotherapy alone in reestablishing not only the number but also the function of BM SLAM + cells allowing them to outcompete leukemic cells during the relapse process. Unfortunately, even though NOS2 is the preferential target of available NO inhibitors, the mechanisms of NOS3 regulation and NO production have not been clarified yet. Several preclinical studies have suggested that the most promising strategy leading to NO inhibition might rely on the targeting of NOS gene regulators.

## 6. Prevention of Immune Escape

HSCT, which remains the only curative treatment for most AML patients, can be considered an immunological procedure since it causes the potential eradication of recipient LSCs through various immunological mechanisms (i.e., activation of donor T lymphocytes and natural killer (NK) cells and production of an inflammatory milieu) that constitute the so-called graft-versus-leukemia (GVL) effect. Peptides presented by self-HLA are recognized as targets by T cells, whereas activation of NK cells depends on the absence or downregulation of self-HLA class I molecules. These two different types of recognition have evolved as complementary strategies to ensure immunological recognition of virus-infected cells in which downregulation of self-HLA class I molecules represents an important mechanism to avoid immunological recognition by virus-specific T cells and promote viral persistence [89, 90]. However, LSCs may effectively escape these immunological mechanisms by altering the expression of target genes and acquiring new selective mutations that eventually determine a posttransplant relapse, still one of the major transplant complications. Currently, the elimination of HLA alleles without the reduction of the overall expression levels of HLA class I molecules achieved through a copy-neutral loss of heterozygosity has been identified as a mechanism of relapse in about one-third of HSCTs, especially those from haploidentical donors, while the downexpression of HLA class II molecules is one of the principal mechanisms of relapse in both HLA-matched and HLA-mismatched HSCTs. Accordingly, recent studies have shown that a higher expression of HLA class II molecules predicts a better prognosis. Currently, there are no therapeutic strategies that can counteract the downexpression of HLA molecules. However, from a clinical point of view, this information explains why the infusion of donor lymphocytes should be avoided in posttransplant relapse with genomic HLA loss, as the infusion of these donor cells might be associated with a less effective GvL effect and may be associated with the development of GVHD.

In addition, LSCs can upregulate the expression of coinhibitory molecules in order to block T-cell activation [91]. Physiologically, the interaction between these molecules (CD80/86 for CTLA-4 and PD-ligand-1/2 (PD-L1/2) for PD-1/2) expressed on antigen-presenting cells (APCs) and various immune-checkpoint molecules (inhibitory molecules (e.g., T lymphocyte-associated antigen-4 (CTLA-4) and programmed death-1 (PD-1)) expressed on activated T cells) leads to controlled T-cell inhibition, which is of primary importance to prevent immune-mediated diseases. Data from several clinical trials support the main role of this T-cell inhibition in AML relapse, in both the transplant and the nontransplant setting. AML patients who relapse after allogenic HSCT present a deregulated expression of PD-L1 and other immune-regulatory molecules (e.g., B7-H3 and CD155/PVRL2). In these patients, various clinical trials have revealed the clinical effectiveness of CTLA-4 blockage in disease control [92–94]. The role of PD-L1 expression has also been shown in JAK2^V617F^ myeloproliferative neoplasms. In these disorders, STAT3 and STAT5 phosphorylation by the oncogene JAK2^V617F^ enhances PD-L1 expression by increasing PD-L1 promoter activity. T cells that interact with these JAK2^V617F^ mutant cells have reduced cell-cycle progression and metabolic activity, explaining why these myeloid cells can escape immune responses [95, 96]. A similar mechanism has also been demonstrated in Myc-driven lymphomas. In these neoplasms, the oncogenic activity of Myc leads to PD-L1 and CD47 overexpression. CD47 is a transmembrane protein that by binding to signal regulatory protein-*α* (SIRP*α*) expressed on APCs limits their activity and blocks their antigen uptake [97]. In these neoplastic cells, Myc inactivation leads to a reduction in PD-L1 and CD47 expression and reverses their phenotype [98]. However, retrospective studies have shown that the administration of anti-PD-L1 in patients with Hodgkin's lymphoma relapsed after allogenic HSCT is associated with high GVHD rates, a potential limitation of immune-checkpoint inhibition in the transplant setting [99]. A higher incidence of both acute and chronic GVHDs has also been reported in other studies that used higher anti-PD-L1 dosages. Ipilimumab is an anti-CTLA-4 antibody whose safety and efficacy were tested in a phase 1/1b multicenter study that enrolled twenty-eight patients with hematological neoplasms relapsed after allogenic HSCT. The overall response rate was 32%, including 23% complete remissions, and responses were long-lasting [94]. In patients who responded to ipilimumab, tissue analysis revealed higher CD8^+^ T-cell and lower Treg counts. Thus, further studies with ipilimumab in AML relapsed after transplant are currently ongoing to establish the effectiveness of this therapeutic strategy in preventing posttransplant immune escape.

Another mechanism of LSCs to evade immune control relies on the production of anti-inflammatory cytokines that create an anti-inflammatory microenvironment able to block the immune response by hindering leukemic cell recognition and destruction. Several studies performed in the nontransplant setting have shown that CML cells can produce IL-4 and TGF-*β* that reduce the expression of MHC class II molecules, an event that renders leukemic cells less immunogenic [100–103]. IL-4 and IL-10 are also secreted by AML and chronic lymphocytic leukemia (CLL) cells, and these cytokines support the CLL immune escape program [104, 105]. Proinflammatory cytokines, including interferon-*γ* (IFN-*γ*), IL-15, IL-1*β*, and granulocyte colony-stimulating factor (G-CSF), are produced by normal myeloid and lymphoid progenitors and improve leukemic cell recognition and immune cell activation. Therefore, it is reasonable for leukemic cells to block the production of these proinflammatory cytokines, as demonstrated by laboratory and clinical data. In a clinical study performed in 393 patients with B-lineage ALL, lower IFN-*γ* levels were associated with a younger age at diagnosis and a high-risk profile defined by prednisone response, cytological remission, and minimal residual disease. This finding supports the role of IFN-*γ* in immunosurveillance and its effect on the early response to steroid therapy [106]. IL-15 is produced by healthy myeloid progenitors and promotes leukemia control by stimulating the generation of human memory stem T cells from naive precursors and by expanding NK cells, thereby boosting the alloimmune effect to eliminate residual LSCs after allogenic HSCT [107–110]. IL-15 also enhances T-cell antitumor activity by reprogramming their mitochondrial metabolism [111–113]. Based on these data, various studies have employed IL-15 to eliminate residual leukemic cells after allogenic HSCT [109]. Apart from HSCT, IL-15 secretion by healthy myeloid precursors stimulates AML cell recognition and elimination by CD8^+^ T cells and NK cells. More importantly, AML cells carrying the internal tandem duplication of the FLT3 gene (FLT3-ITD) are able to block IL-15 production. In animal studies, FLT3 inhibition together with T-cell transfer promoted AML cell elimination and helped achieve long-term disease control [111]. IL-15 production stimulated by sorafenib and midostaurin enhances the immunogenicity of leukemic cells and the activation of T cells. This result supports the role of FLT3 activity in the regulation of IL-15 secretion.

Moreover, it can be speculated that other mechanisms of immune escape may rely on the block of several proinflammatory cytokines, such as G-CSF (which is involved in APC maturation) and IL-1*β*. Further studies are needed to clarify the mechanisms by which these inflammatory cytokines help the immune system recognize and eliminate leukemic cells in order to develop therapeutic strategies. However, it is known that these cells can also evade immune surveillance by producing various immunosuppressive enzymes. One of these, indoleamine 2,3-dioxygenase 1 (IDO1), is responsible for the first step of tryptophan degradation and subsequent kynurenine production. Both the lack of tryptophan and the presence of kynurenine negatively affect T-cell functions and reprogram Treg activity [114]. IDO1 is expressed by leukemic cells, and its production has been linked to an unfavourable prognosis [115]. Arginase, another enzyme produced by AML cells, is required for the degradation of arginine that is needed for T-cell proliferation and polarization of monocytes toward an inflammatory M1-like phenotype. In laboratory studies, T-cell proliferation and NK-cell proliferation were inhibited when AML cell supernatants were added to lymphocytes cultures [116, 117]. Ectonucleotidase (CD73) is an enzyme required for enzymatic cleavage of adenosine monophosphate (AMP), which results in an adenosine with an immunosuppressive effect. Thus, CD73 inhibition could be a promising therapeutic target as it might enhance leukemia control. CD39, another ectonucleotidase that produces adenosine diphosphate (ADP) and AMP from adenosine triphosphate (ATP), might also represent a logical target to prevent the immunosuppressive activity of AML cells [118, 119].

## 7. Conclusions

AML treatment has remained unchanged over the past twenty-five years and is frequently associated with dismal outcomes. This has not changed with the development of molecular drugs that are mutation specific, as they do not address the genetic heterogeneity of the disease. Thus, there is a need for the development of novel treatments that target the cellular and molecular mechanisms controlling dynamic AML-niche interactions and resolve niche-mediated drug resistance. Current studies have continued to highlight the role of the niche in AML development and progression, and potential targets of niche-directed treatments are now starting to emerge. AML xenograft models have revealed that severe vascular BM damage consisting of increased vascular leakiness occurs as the primary AML-induced niche alteration and leads to mobilization of healthy HSCs to the periphery. This experimental finding may seriously limit the success of allo-HSCT by reducing the outcompetition effect of patient residual and donor-derived HSCs on the AML cell population. Therefore, studies aimed at keeping HSCs in their BM niches are warranted [120]. This goal might be achieved by considering studies that aim to reveal very specific requirements of the neoplastic cell to alter their microenvironments. For example, a current study has suggested that the main determinant of LSC or HSC physical localization might be their metabolic status (i.e., glycolytic level) [121]. Using a metabolic imaging system with a highly responsive genetically encoded metabolic sensor (SoNar), this study identified pyruvate dehydrogenase kinase 2 (PDK2) as the enzyme that fine tunes glycolysis, homing, and symmetric division of LSCs. SoNar-high cells, which prefer homing to the endosteal niche and symmetric division to maintain their leukemogenic activities, are more glycolytic, enriched for higher LSC frequency, and develop leukemia much faster than SoNar-low counterparts. Thus, these recent findings have pinpointed novel relevant niche alterations that are expected to be the basis for the development of innovative strategies aimed at eradicating LSCs and sparing normal HSCs.
